# Discourse Measures to Differentiate Between Mild Cognitive Impairment and Healthy Aging

**DOI:** 10.3389/fnagi.2019.00221

**Published:** 2019-08-21

**Authors:** Bo Seon Kim, Yong Bum Kim, HyangHee Kim

**Affiliations:** ^1^Graduate Program in Speech-Language Pathology, Yonsei University, Seoul, South Korea; ^2^Department of Neurology, Kangbuk Samsung Hospital, Sungkyunkwan University School of Medicine, Seoul, South Korea; ^3^Department and Research Institute of Rehabilitation Medicine, Yonsei University College of Medicine, Seoul, South Korea

**Keywords:** mild cognitive impairment, discourse, measures, cognition, language impairment

## Abstract

Although subtle discourse declines in people with mild cognitive impairment (MCI) have been reported, heterogeneous measures and tasks among the MCI discourse studies have yielded widely varying outcomes. The present study aimed to first, identify discourse measures that aid the differentiation among people with amnestic MCI (aMCI), people with non-amnestic MCI (naMCI), and cognitively healthy control (HC) participants, and second, delineate the cognitive functions related to such discourse measures. Three discourse tasks (an episodic narrative, a planning task, and a picture description) were performed by 30 aMCI, 22 naMCI, and 21 HC participants. Samples were analyzed using six categories of 15 measures, namely coherence, cohesion, proposition, grammaticality, lexicality, and fluency. The statistical analyses included (1) a multivariate analysis of variance for group comparison; (2) binary simple logistic regression and receiver operating characteristic curve analysis for differentiation between two groups; (3) binary multiple logistic regression for being diagnosed with naMCI or aMCI with the minimum number of predictors; and (4) Pearson correlation analysis for identifying the cognitive functions associated with the discourse measures. The proportion of cohesive words and propositional density in aMCI participants were worse than those in naMCI participants. Global coherence, the proportion of cohesive words, and the proportion of dysfluencies and pauses in naMCI participants were lower than those in the HC participants. Global and local coherence and the proportion of cohesive words, cohesive ties per utterances, propositional density, and dysfluencies and pauses in aMCI participants were worse than those in the HC participants. The aforementioned measures were demonstrated to be effective predictors for classifying groups by receiver operating characteristic curve analysis. In addition, the proportions of cohesive words and pauses were common discourse measures for differentiation between naMCI and HC participants or between aMCI and HC participants using binary multiple logistic regression. According to the correlation analysis, memory and executive functions are needed for coherent, cohesive, and efficient discourse productions in MCI. The detailed description of discourse performances in this study will aid the characterization of the declined language abilities of MCI participants and also the understanding of the cognitive functions involved in discourse performance in MCI.

## Introduction

Older individuals with complaints with regard to their cognitive function may undergo neuropsychological tests to determine which functions are impaired. Older individuals who have not been diagnosed with dementia but have lower cognitive function than do normal adults are considered to have mild cognitive impairment (MCI). MCI can be categorized into subtypes, which are determined by the level of performance in each domain of the neuropsychological test (Rao et al., 2018). Although most MCI studies have focused on amnestic MCI (aMCI), wherein the person has memory impairment, non-amnestic MCI (naMCI), wherein a person has other cognitive impairments apart from memory, also exists. In addition, MCI can be divided into four subtypes based on the cognitive domains that are impaired. If only memory impairment is present, the individual has single domain aMCI (sd-aMCI). If there are other non-memory cognitive impairments such as language, attention/executive function, or visuospatial abilities, the individual has multiple domain aMCI (md-aMCI). In case of naMCI, if only one cognitive domain is impaired apart from memory, the individual has single domain naMCI (sd-naMCI), and if more than one domain is impaired, the individual has multiple domain naMCI (md-naMCI). These subtypes may have prognostic utility in that aMCI is suggested to develop into Alzheimer’s disease (AD), and naMCI is suggested to develop into frontotemporal dementia and dementia with Lewy bodies (Petersen, 2003, 2004). In a recent meta-analysis of 33 MCI studies, among the four subtypes, sd-naMCI prevalence was the highest in community samples and md-aMCI prevalence was the highest in clinic samples. Overall, the prevalence of aMCI was higher in clinic (88%) than in community studies (51%) and that of sd-MCI was higher in community (66%) than in clinic samples (41%) (Oltra-Cucarella et al., 2018). For the diagnosis of each MCI subtype, performance on neuropsychological tests in four areas, namely memory, language, visuospatial, or frontal functions (or attention/execution) is below −1.5 standard deviation (SD) (Ferman et al., 2013; Oltra-Cucarella et al., 2018) or −1.0 SD (Busse et al., 2006; Kim et al., 2014). Both md-aMCI and sd-aMCI were more likely to convert to AD and sd-naMCI and md-naMCI were less likely to convert to AD (Oltra-Cucarella et al., 2018). However, naMCI was reported to have a tendency to convert to non-AD dementia (e.g., dementia with Lewy bodies) (Busse et al., 2006; Ferman et al., 2013).

Discourse can be defined as a set of utterances aimed at conveying a message among interlocutors, and may be considered the most elaborative of linguistic activities (Kent, 2003). Since discourse is a multilevel object of study, its production implies the simultaneous activation of various components, such as phonology, lexicon, syntax, the elaboration of macrostructure, the establishment of cohesion, and coherence links (Joanette and Brownell, 2012). However, discourse tasks have typically been selected as per the dementia types and discourse measures that investigators want to study. For example, participants with mild AD could be differentiated from the cognitively healthy control (HC) using a complex picture description task rather than a simple task (Forbes-McKay and Venneri, 2005). Participants with Parkinson’s disease with dementia are reported to have reduced basal ganglia function due to dopamine depletion of the substantia nigra and also reduced frontal lobe function; thus, they have reduced executive function and working memory (Ash et al., 2012; Assala and Ghikab, 2013).

In fact, studies that analyzed the discourse of people with dementia or with impaired brain lesions rarely included a comprehensive cognitive test. However, some studies suggest that memory and executive functions are related to the performance of discourse. When amnestic patients were asked to describe past and future events, both coherence and cohesion of the patient group were significantly lower than those of the normal group (Race et al., 2015). In addition, executive function was correlated with performance of global coherence by error analysis in the “Picnic Scene” of the Western Aphasia Battery (Kertesz, 1982) and the two stories about sequential pictures for patients with traumatic brain injury (TBI) (Marini et al., 2014). Theme maintenance analyzed with “Frog, Where Are You?”(Mayer, 1969) in individuals with brain lesions was reported to correlate with planning and organization (e.g., the performance of letter fluency tasks) (Ash et al., 2014). A correlation between global coherence (three-point rating scale) and attention and processing speed was also reported in stroke patients (Rogalski et al., 2010). However, to our knowledge, few studies have examined the relationship between comprehensive cognitive functions and the performance of discourse that measures macro- and micro-aspects of early dementia patients or high-risk dementia patients.

In addition, many discourse studies restricted the analysis to a small number of measures in one genre produced by a small number of participants, which was due to the complexity and time-consuming nature of the analysis as well as the relative scarcity of adequate discourse processing theories and models (Sherratt, 2007). To our knowledge, the models of discourse processing for brain damaged participants comprise the text-processing theory (Kintsch and Van Dijk, 1978; Joanette and Brownell, 2012), the multi-layered discourse processing model (Duong et al., 2005; Sherratt, 2007; Joanette and Brownell, 2012), and the Structure Building Framework (Gernsbacher, 1990). According to the text-processing theory, macroprocessing enables the speaker/hearer to deal primarily with the most important ideas in a text. Microprocessing, however, deals with individual propositions and their relationships as conveyed by various syntactic and stylistic cohesion devices such as reference relations, attributive and adverbal specifications, changes in word order, paraphrases, repetitions, etc. (Kintsch and Van Dijk, 1978; Joanette and Brownell, 2012). In brief, macroprocesses generalize and summarize the contents of the micropropositions (Kintsch and Van Dijk, 1978).

The multi-layered discourse processing model is more stratified than the text-processing theory. First, processes operating on conceptual networks consisting of frame generation, which generate one or more conceptual frames and act as organizing principles for the discourse. Second, processes of propositions generate specific propositions regarding selected information and bind them together into units such that they can be understood in terms of a connected semantic unit. Third, the linguistic encoding of proposition chunks occurs based on syntactic, lexical, and morphological linguistic knowledge (Sherratt, 2007; Joanette and Brownell, 2012; Sherratt and Bryan, 2012).

The Structure Building Framework (Gernsbacher, 1990) is a model designed to explain discourse comprehension. However, it has been used to interpret discourse production deficits due to communicative impairments. According to this model, there are three steps required to build coherent mental representations or structures of the information being processed. First, a foundation should be laid for a mental structure. Second, the memory cells activated by the incoming stimuli are mapped based on the foundation. Third, by suppressing irrelevant information or enhancing relevant information, the shift to a new substructure occurs. In schizophrenic participants, ineffective suppression mechanisms, difficulty in laying the foundation, and frequent shifts result in disordered (i.e., verbose or impoverished) discourse (Gernsbacher et al., 1999). In addition, impaired discourse production in participants with traumatic brain injury was found to result from decreased cognitive functions concerning the laying of the foundations or mapping (Coelho et al., 2013; Marini et al., 2017).

Although a few studies have analyzed discourse production in prodromal or minimal AD (Forbes et al., 2001; Forbes-McKay and Venneri, 2005; Ahmed et al., 2013a, b), seven studies have investigated the characteristics of discourse of participants with MCI and one study investigated the characteristics of discourse of participants with subclinical MCI from 2001 to 2018. In previous studies using a picture description task of “Cookie Theft” (Goodglass and Kaplan, 1983), the number of semantic units of MCI participants did not differ from that of the healthy controls (Bschor et al., 2001). There were significant differences in semantic and fluency factors (Mueller et al., 2018). This difference seems to be caused by differences in the level of detail of the discourse analysis (Mueller et al., 2017). In addition, in the task that involved listening to and retelling a biographical narrative, the discourse of the MCI group was more impaired with regard to the gist (i.e., summary, main idea, and interpretive statements) and level of detail (i.e., recall and recognition of details) of discourse processing (Chapman et al., 2002). In a recent study (Drummond et al., 2015) using a car accident task involving seven sequential pictures (Duong et al., 2005), discourse between the aMCI and healthy control groups differed with regard to the speech effectiveness index (i.e., the number of words in macropropositions), and the aMCI participants yielded more irrelevant micropropositions than did the control group. Finally, among the four studies that compared the discourse performance of MCI and healthy groups using the task of “Trip to New York” (Fleming and Harris, 2008, 2009; Harris et al., 2008; Fleming, 2014), which includes the characteristics of narrative and procedural speech, two studies reported discourse impairment in the MCI group (Fleming and Harris, 2008; Harris et al., 2008). The former study reported that the discourse of MCI participants was impaired in length and quality compared to those of the control group, and the latter study reported that less thematic information was produced in the MCI group than in the control group.

Although studies on dementia and MCI discourse have been conducted in this manner, several limitations exist. First, in the existing MCI discourse studies, it is difficult to interpret how discourse in MCI participants differs from that of the HC participants in according to each genre of discourse. Although the discourse studies of MCI have yielded promising results in measuring subtle linguistic impairments, there is a limit to judging the result as a discourse characteristic of general connected speech in MCI using only one genre. Second, most pathological discourse studies have not organized results regarding stratified discourse processing. Systematic presentations of discourse performance with model-based measures will benefit investigators and clinicians concerning MCI participants with respect to diagnosis and intervention. Third, the type of MCI was not mentioned (Bschor et al., 2001; Fleming and Harris, 2008; Harris et al., 2008) or only aMCI was included (Chapman et al., 2002; Fleming and Harris, 2009; Drummond et al., 2015) or aMCI and naMCI were both included as MCI participants (Fleming, 2014). As MCI discourse studies attempt to measure subtle linguistic impairments, there may be other discordance patterns between naMCI and aMCI.

Therefore, the purposes of this study were two-fold. First, the differences among the aMCI, naMCI, and HC groups were examined by applying various measures from discourse extracted from various tasks. Second, we investigated the cognitive functions associated with each measure of discourse. This study investigated the differences in discourse performance per the subtypes of MCI (i.e., aMCI and naMCI). The two subtypes of MCI are divided based on whether memory impairment was present. Thus, we hypothesized that aMCI participants would exhibit deficits at the level of insertion and integration of semantic information due to memory problems, and naMCI participants would experience greater difficulty at the syntactic level rather than at the conceptual and propositional level due to declined linguistic structural abilities or impaired attentional/executive function. Therefore, discourse in aMCI might comprise impairments in measures of coherence, cohesion, propositional density, and fluency. To our knowledge, impairments in the discourse performance of naMCI participants have not been previously investigated. However, their declined frontal executive functions or attention might impact lower level syntactic or fluency abilities.

## Materials and Methods

### Participants

The HC participants in this study were part of a large study aimed at developing the protocol of language and cognitive function tests in older adults (IRB#: 1-2011-0061). Initially, 113 older participants (age < 60 years) performed language tests and three discourse tasks in this study from May 2012 to August 2012 in Seoul, Daejeon, and Busan in South Korea. They were recruited from the community welfare center in Seoul and the senior citizen center in Daejeon and Busan. The questionnaires and language and cognitive function tests for this study were performed by graduate students of language pathology. The participants also answered pre-evaluation questions to confirm adequate hearing and vision for testing and a questionnaire for excluding participants with a history of or current neurological or psychiatric disorders. Three items were used for adequate vision and two for adequate hearing. The three items of the visual test were configured to present small letters sequentially and the participants were requested to read them. The hearing test items were composed of hearing two sentences, “Pick the right (left) open circle,” and the participants were requested to select an appropriate one. The questionnaire was used to exclude participants who were diagnosed, operated upon, or hospitalized due to the following diseases: stroke, epilepsy, Parkinson’s disease, multiple cerebral sclerosis, Huntington’s disease, encephalitis, MCI/dementia, psychosis, and depression. Initially, 54 older participants whose education was <6 years were excluded, because the efficiency of spontaneous speech (i.e., the percentage of correct information units) and the number of morphemes differed between older participants with >6 years of education and those with <6 years (Lee and Kim, 2001; Cheon, 2011). In addition, 11 older participants who reported that their literate ability was not good (i.e., they cannot read and write letters) were excluded, because this study also investigated the production of complex sentence structure via morpheme analysis (Lee and Kim, 2001). Finally, among the remaining 48 older participants, 18 with scores on the Korean version of the MMSE (K-MMSE) that were below the cut-off point were excluded, taking into account age and years of education (Kang, 2006). However, because the cut-off score on the MMSE has been reported as 25 or 26 for MCI, 9 participants with scores below 26 on the K-MMSE were also excluded, resulting in 21 participants.

From February 2016 to August 2016, 53 participants with aMCI and 29 participants with naMCI were recruited from the Department of Neurology in Kangbuk Samsung Hospital in Seoul (IRB#: KBSMC 2015-12-045). All participants with MCI were diagnosed by a neurologist or a neuropsychologist at the department. The participants with MCI consisted of those who visited the hospital with subjective cognitive complaints or because of suggestions by their children, spouses, or relatives; no inpatients were included. The criteria for aMCI were follows: (1) participants with complaints of memory loss; (2) participants with delayed recall scores on the Seoul Verbal Learning Test-Elderly’s version (SVLT) that decreased to below −1 SD considering age and education, a Clinical Dementia Rating (CDR) score of 0.5, and a score of 0.5 or 1 on the memory item; (3) absence of significant dysfunction of everyday life; (4) dementia not diagnosed by a neurologist or a neuropsychologist; and (5) written consent to participate in the study. The criteria for naMCI were follows: (1) participant complaints of cognitive function decline or cognitive decline demonstrated by objective tests; (2) presence of objective cognitive impairment, indicated by one or more of the following: (a) decline in visuospatial function [<−1 SD on the Rey Complex Figure Test (RCFT)], (b) impaired language ability (<−1 SD on the Boston Naming Test), (c) impaired frontal/executive function (motor executive function, phonemic and semantic verbal fluency test, and Stroop’s test), with impaired frontal/executive function defined as impairment in at least two of the three groups (Kim et al., 2014); (3) criteria (3) to (5) as described for aMCI above. Of the aMCI participants, 14 were excluded due to having <6 years of education, six were excluded because they were aged <60 years, two were excluded because they refused to complete the test, and one was excluded due to illiteracy. Among the 29 naMCI participants, six were excluded due to having <6 years of education and one was excluded because she was aged <60 years. Finally, 30 aMCI and 22 naMCI participants were included in this study. This study was approved by the Institutional Review Boards of the Severance Hospital and the Kangbuk Samsung Hospital. All participants gave written informed consent in accordance with the Declaration of Helsinki.

The demographic information of the MCI participants and HC participants is presented in Table 1. The groups did not differ in age (*F* = 2.087, *p* > 0.05), gender (χ^2^ = 3.111, *p* > 0.05), mean years of education (*F* = 1.691, *p* > 0.05), K-MMSE score (*F* = 1.357, *p* > 0.05), and the 15 item short version of Geriatric Depression Scale (GDS) (*F* = 3.319, *p* > 0.05).

**TABLE 1:** Demographic information of MCI and healthy control participants.

	**aMCI (*n* = 30)**	**naMCI (*n* = 22)**	**HC (*n* = 21)**	***P***
Age (years)	73.80 ± 6.41	70.09 ± 6.27	71.90 ± 6.84	0.132
Gender (M:F)	11:19	6:16	3:18	0.211
Education (years)	10.40 ± 3.77	9.55 ± 3.90	8.52 ± 2.91	0.192
K-MMSE	26.63 ± 2.19	27.45 ± 1.95	27.19 ± 0.98	0.264
GDS	6.90 ± 3.92	5.45 ± 3.88	4.29 ± 2.76	0.092

*Mean ± SD. aMCI, amnestic mild cognitive impairment; naMCI, non-amnestic MCI; HC, healthy control; K-MMSE, the Korean version of the Mini-Mental State Examination; GDS, the 15-item short version of Geriatric Depression Scale.*

### Discourse Tasks

The three discourse test materials were comprised of two tasks that were presented with oral instructions and one task that was presented with oral instructions and a picture stimulus (Kim et al., 2009). A narrative of a biographical experience (about raising children), a picture description, and the “Trip to Jeju Island” task which includes narrative and expository characteristics adopted and revised from the “Trip to New York” task (Fleming and Harris, 2008, 2009; Harris et al., 2008; Fleming, 2014) were conducted in this order. In addition, all discourse tasks were audio recorded and transcribed orthographically and verbatim with Microsoft word 2016 checking word spacing, typographical errors, and spelling errors.

### Procedures

After pre-evaluation questions and the demographic information questionnaire, a K-MMSE was performed. The three discourse tasks were performed in approximately 5-10 min. In the HC participants, after the K-MMSE and discourse tasks, the K-BNT-15 (Kim and Kim, 2013) and 30 s verbal fluency test (animal naming) (Kim et al., 2011) were administered. Participants with MCI answered the same questionnaires, K-BNT-15, and verbal fluency test, as the HC participants. Additionally, the Seoul Neuropsychological Screening Battery 2nd Edition (SNSB) (Kang et al., 2012) procedure was performed. However, the HC group did not perform SNSB as they received another cognitive test that was being studied (Lee and Kim, 2016).

### Neuropsychological Test

The SNSB has been shown as a useful tool for predicting cognitive function decline in patients with early dementia (Kim et al., 2017) as well as establishing the correlation between cognitive functions and brain imaging in individuals with dementia (Ahn et al., 2011; Lee et al., 2013). In this study, cognitive function tests (i.e., parts of SNSB) were included as follows: the attention tests comprised the Digit Span Test backward and forward; the memory tests comprised the Immediate Recall and Delayed Recall of the SVLT and the RCFT; and frontal/executive functions were tested using semantic verbal fluency tests (animal and supermarket), phonemic verbal fluency tests (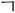, 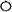, 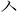), and a Word Reading and Color Reading of Korean-Color Word Stroop Test (CWST). Table 2 presents the neuropsychological test results of the aMCI and the naMCI participants. There were significant group differences between the aMCI and the naMCI participants in language (15-item Korean version of the Boston Naming Test [K-BNT]-15: *p* < 0.001), all memory tests (*p* < 0.001 and *p* < 0.05), and executive functions with semantic verbal fluency (*p* < 0.05) and Color Word Stroop Test of color reading (*p* < 0.05). The scores of the aMCI participants in these previously mentioned cognitive and language functions were lower than those of the naMCI participants.

**TABLE 2:** Comparison of the neuropsychological test scores between aMCI and naMCI.

**Cognition**	**Test**		**aMCI**	**naMCI**	***p***
Attention	Digit span test				
	Forward		5.80 ± 1.10	6.45 ± 1.47	0.087
	Backward		3.47 ± 0.57	3.64 ± 1.26	0.560
Language	K-BNT-15		11.03 ± 3.01	13.95 ± 1.62	<0.001
Visuospatial function	Rey complex figure test				
	Score		32.00 ± 4.50	32.61 ± 3.91	0.610
	Time		304.57 ± 135.19	236.59 ± 129.31	0.074
Memory	Seoul verbal learning test				
	Immediate recall		13.73 ± 2.92	18.68 ± 5.22	<0.001
	Delayed recall		1.00 ± 1.49	5.50 ± 1.54	<0.001
	Rey complex figure test				
	Immediate recall		6.22 ± 5.50	13.09 ± 7.62	<0.001
	Delayed recall		5.62 ± 6.59	12.36 ± 6.99	0.001
Executive function	Semantic verbal fluency		12.40 ± 4.11	16.16 ± 3.38	0.001
	Phonemic verbal fluency		5.02 ± 2.55	6.73 ± 4.20	0.101
	Color word stroop test				
	Word reading	Correct response	109.80 ± 7.17	110.91 ± 2.51	0.491
		Time	84.90 ± 19.60	82.18 ± 24.14	0.656
		Time per item	0.78 ± 0.22	0.74 ± 0.23	0.551
	Color reading	Correct response	62.30 ± 28.55	80.64 ± 22.14	0.015
		Time	118.67 ± 5.88	118.73 ± 5.54	0.970
		Time per item	3.72 ± 8.13	1.51 ± 0.41	0.149

*aMCI, amnestic mild cognitive impairment; naMCI, non-amnestic mild cognitive impairment; K-BNT-15, the 15-item Korean version of the Boston Naming Test.*

### Discourse Measures

According to the stratified discourse processing model, discourse measures can be categorized into six measuring areas, and these belong to each processing level (Sherratt, 2007; Joanette and Brownell, 2012; Sherratt and Bryan, 2012; Kim and Kim, 2015). The specific measures and criteria of the six measuring areas were as follows:

(1) Coherence: Global and local coherence were scored with 4-point and 2-point rating scales, respectively. Utterances (Shewan, 1988; Kim et al., 1998, 2006; Kwon et al., 1998; Lee and Kim, 2001; Marini et al., 2011; Andreetta et al., 2012) were used as the unit for scoring both coherence scales. Utterances are segmented by grammatical criteria (e.g., an ending morpheme, a conjunction, or a conjunctive adverb), semantic criteria (e.g., a component that is linked to a preceding utterance in included in a preceding one), and phonological criteria [e.g., a falling intonation or a pause more than 2 s (except for pauses due to word-finding difficulties)]. Global coherence refers to the relationship between the main topic and the meaning or content of the utterances of discourse to be analyzed, that is, how utterances maintain the overall topic (Wright et al., 2014; Kim et al., 2018). The measures that have been used in global coherence studies are the mean scores from utterance scores with 4-point or 5-point rating scales (Glosser and Deser, 1991; Coelho et al., 2012; Le et al., 2014; Wright et al., 2014). This study used four-point rating scales for global coherence. One point was given to utterances that were entirely irrelevant to the topic, two for those only remotely related to the topic, three for those lacking specific information about a given topic, and four for those containing specific information relating to a given topic (Kim et al., 2018). To score local coherence, a two-point rating scale was used to capture each utterance’s appropriateness or lack of local coherence. Local coherence included relationships of continuation, repetition, elaboration, subordination, or coordination with the topic in the immediately preceding utterance (Glosser and Deser, 1991).
(2) Cohesion: Words of closed-class lexical cohesion (i.e., references, such as personal, demonstrative pronouns, and determiners) and open class lexical cohesion (i.e., repetitional and synonymous phrases, superordinate designate and subordinate exemplar) were counted and divided by the total number of words (Glosser and Deser, 1991; Caspari and Parkinson, 2000). Additionally, conjunctions including temporal and causal cohesion were counted and divided by the number of words. The morphemes used as cohesive ties were also analyzed using the GeulJabi morpheme program. Connective endings and assistive endings were tallied by the program and the percentage of cohesive morphemes was calculated by dividing the number of cohesive morphemes by the total number of morphemes. In addition, cohesive ties, which are the sum of cohesive words and morphemes, were divided by the number of utterances in order to investigate the frequency of cohesive ties in each utterance.
(3) Proposition: The number of propositions and that of novel propositions were counted, and the number of novel propositions per propositions (i.e., the propositional density) was recorded (Joanette and Brownell, 2012; Robinson, 2013; Law et al., 2015). Novel propositions in this study are defined as related propositions to the topic or the stimulus aside from repetition and entirely unrelated propositions. To segment utterances into propositions, utterances were divided into propositions using part-of-speech tagging by the GeulJabi morpheme program (Brown et al., 2008; Bryant et al., 2013).
(4) Grammaticality: Measures for syntactic abilities were adapted from Frederiksen et al. (2012). Parts-of-speech units (i.e., nouns and verbs), proportion of clausal endings, and the mean length of utterances were calculated semi-automatically by the GeulJabi morpheme analysis program. The clausal endings in this study are defined as the number of connective and transitional endings calculated by the GeulJabi morpheme analysis program. We excluded the number of connective endings for serial verbs (Lee et al., 2012). The proportion of clausal endings was calculated by the sum of the connective and transitional endings divided by the total number of morphemes. The total number of morphemes was divided by the number of utterances to determine the mean length of utterances. Syntactic measures (i.e., parts-of-speech units, proportion of clausal endings, and mean length of utterances) were analyzed using the GeulJabi morpheme analysis program. Although the GeulJabi morpheme analysis program was used for calculating the number of endings and morphemes, this program resulted in errors such as repetitional counts of endings and the inclusion of punctuation marks, commas, or question marks in the number of morphemes. Therefore, the results of the program were also manually reviewed and coded.
(5) Lexicality: Measures for lexical analyses were also adapted from Frederiksen et al. (2012), Joanette and Brownell (2012). The number of different words was calculated using the GeulJabi morpheme analysis program, which is the number of total parts-of-speech units (Ok, 2011). Types of parts-of-speech units consist of nouns, pronouns, numerals, unconjugated adjectives, adverbs, interjections, verbs, and adjectives. Although there are nine parts-of-speech units in the Korean language, including the previously mentioned eight constituents with particles, we excluded particles because these have only morphosyntactic features (Lee, 2010). The number of different words was divided by the total number of words to determine the type token ratio. To exclude the automatic program errors, each parts-of-speech unit was reviewed and compared with transcriptions.
(6) Fluency: Dysfluent words such as fillers, repetitions, revisions, and pauses were counted and divided by the total number of words to calculate the proportion of dysfluencies (Lim and Hwang, 2009; Lee and Lee, 2014). The speech rate for which the total number of words was divided by the total talking time in s was also measured (Gayraud et al., 2011). A pause in the study was measured in seconds taken to initiate discourse; the pause length being more than 2 s between and within utterances (Singh et al., 2001). The proportion of pauses was also calculated by dividing the total time of pauses in seconds by the total talking time.

### Reliability

Intra-rater and inter-rater reliability of transcriptions were performed on 10% of the sample (i.e., 24 transcripts of eight participants with three HC participants and five MCI participants). The researcher re-transcribed 24 audio-recording samples of eight participants. The intra-rater reliability with word-by-word agreements was 95.18%. For the inter-rater reliability, a speech-language pathologist with >5 years’ experience transcribed 24 audio-recording samples and the inter-rater reliability was 95.00% with word-by-word agreements.

Point-to-point agreements were evaluated for inter-rater and intra-rater reliability in segmenting utterances and scoring global coherence. Of the total discourse materials, 10%, which amounted to 24 transcripts from eight participants (i.e., four HC participants and four MCI participants) were used for reliability analysis. For utterance segmentation, the intra-rater reliability was 92.31%. For inter-rater reliability of utterance segmentation, a speech-language pathologist with >5 years’ experience segmented the transcripts after reviewing the criteria for utterances and practicing an example with audio recordings. The inter-rater reliability of utterances was 90.68%.

The intra-rater for global coherence was 90.79%. For inter-rater global coherence reliability, the multi-step training protocol (Wright et al., 2013) was conducted, which involved explanations of tasks and scoring methods, reviews of two transcripts with each global coherence score of the utterances, and practices with another two transcripts with explanations. The inter-rater reliability was 91.45% for scoring global coherence.

For grammaticality, the inter-rater reliability and intra-rater reliability were calculated from 9 discourse scripts of three participants (i.e., each from aMCI, naMCI, and HC). The Cronbach’s α-values of the number of morphemes, connective endings, and transitional endings were 0.995, 0.986, and 1.000, respectively in the inter-rater reliability. The Cronbach’s α-values of those in the intra-rater reliability were 0.993, 0.996, and 0.993, respectively.

The Cronbach’s α-values for pauses were also calculated. The Cronbach’s α-value for inter-rater reliability was 0.942 and that for intra-rater reliability was 0.968, calculated from 9 discourse audio records (e.g., one record of aMCI, naMCI, and HC, respectively).

### Statistical Analysis

The average numbers from various measures of the three discourse tasks were used as independent variables. The group differences between the HC participants and the MCI participants were evaluated as follows: (1) a multivariate analysis of variance (MANOVA) was performed to compare the discourse performance among the three groups (aMCI, naMCI, and HC); (2) a binary logistic regression and receiver operating characteristic (ROC) curve for each measure exhibiting a different discourse performance among groups were performed to investigate whether the measures differed among the aMCI participants, naMCI participants, and HC participants; (3) a binary logistic regression analysis for various measures that exhibited group differences was conducted to evaluate the dependent variables (i.e., being diagnosed with naMCI or aMCI) with the minimum number of predictors; and (4) to delineate the cognitive functions that were involved in the discourse measures, Pearson’s correlation coefficient was computed between the discourse measures and cognitive test scores of the aMCI and naMCI participants.

## Results

### Comparison of Discourse Performance

Significant group differences were investigated using a MANCOVA, excluding ten measures among the initial 25 measures that had strong correlations with each other to control for multicollinearity. A Bonferroni correction was performed to adjust for multiple comparisons, which lowered the alpha level to 0.003 (0.05/15). The performances among the three groups differed in global coherence (*F* = 7.946, *p* < 0.001), the proportion of cohesive words (*F* = 16.045, *p* < 0.001), propositional density (*F* = 11.411, *p* < 0.001), the proportion of dysfluencies (*F* = 7.260, *p* < 0.001), and the proportion of pauses (*F* = 9.299, *p* < 0.001) (Table 3).

**TABLE 3:** Comparison of discourse performance in aMCI, naMCI, and healthy control.

**Measures**		**aMCI**	**naMCI**	**HC**	***P***	***Post hoc***
Coherence	Global coherence (S)^∗^	2.72 ± 0.41	2.85 ± 0.27	3.10 ± 0.27	0.001	aMCI, naMCI < HC
	Local coherence (S)	0.85 ± 0.09	0.89 ± 0.08	0.92 ± 0.11	0.032	
Cohesion	Proportion of cohesive words^∗^ (%)	16.15 ± 3.49	19.06 ± 2.45	21.68 ± 4.23	<0.001	aMCI, naMCI < HC, aMCI < naMCI
	Proportion of cohesive morphemes (%)	15.61 ± 4.41	14.89 ± 4.94	18.01 ± 5.62	0.100	
	Cohesive ties per utterance (N)	2.36 ± 0.61	2.56 ± 0.66	2.94 ± 0.80	0.015	
Proposition	Propositional density^∗^ (%)	64.50 ± 11.86	71.64 ± 8.10	78.03 ± 8.94	<0.001	aMCI < HC, aMCI < naMCI
Grammaticality	Proportion of clausal endings (%)	16.41 ± 3.44	15.64 ± 3.95	18.19 ± 4.70	0.104	
	MLU (N)	7.82 ± 1.52	7.94 ± 1.51	7.74 ± 1.16	0.896	
Lexicality	Proportion of nouns (%)	23.86 ± 5.92	26.36 ± 5.18	26.43 ± 6.17	0.190	
	Proportion of verbs (%)	14.18 ± 3.36	13.54 ± 2.4	16.34 ± 7.76	0.145	
	NDW (N)	35.46 ± 15.63	36.36 ± 11.66	33.78 ± 17.08	0.849	
	TTR (N)	0.63 ± 0.06	0.64 ± 0.06	0.63 ± 0.06	0.122	
Fluency	Proportion of dysfluencies^∗^ (%)	16.06 ± 6.62	14.22 ± 4.12	9.88 ± 5.85	0.001	aMCI, naMCI > HC
	Speech rate (s)	1.22 ± 0.31	1.27 ± 0.28	1.33 ± 0.30	0.407	
	Proportion of pause^∗^ (%)	12.73 ± 10.24	11.21 ± 9.35	2.47 ± 4.69	<0.001	aMCI, naMCI > HC

*Mean ± SD. Asterisks (^∗^) indicate discourse measures with significant group differences after Bonferroni correction (*p* < 0.003). (N), number; (s), seconds; (S), score; (%), percentage. aMCI, amnestic mild cognitive impairment; naMCI, non-amnestic mild cognitive impairment; HC, healthy control participants. MLU, mean length of utterance; NDW, number of different word; TTR, type token ratio.*

Per the Bonferroni *post hoc* test results, adjusting for years of education, the discourse measures in the aMCI participants were significantly lower than those of the naMCI participants in the proportion of cohesive words (*p* < 0.011) and propositional density (*p* < 0.041).

The values of the discourse measures for the naMCI participants were significantly lower than those for the HC participants in the global coherence (*p* < 0.048) and the proportion of cohesive words (*p* < 0.046). The discourse measures for the naMCI participants were higher than those for the HC participants in the proportion of dysfluencies (*p* < 0.047) and pauses (*p* < 0.005).

In addition, there were significant group differences between the aMCI participants and the HC participants. Global coherence, the proportion of cohesive words, and propositional density were significantly lower for the aMCI participants than for the HC participants (all three measures: *p* < 0.001). The proportion of dysfluencies and pauses for the aMCI participants was also higher than that for the HC participants (both measures: *p* < 0.001). The scatter plots of the global coherence, proportion of cohesive words, propositional density, and proportion of pauses that had significant differences among the three groups are presented as Supplementary Materials.

**FIGURE 1:**
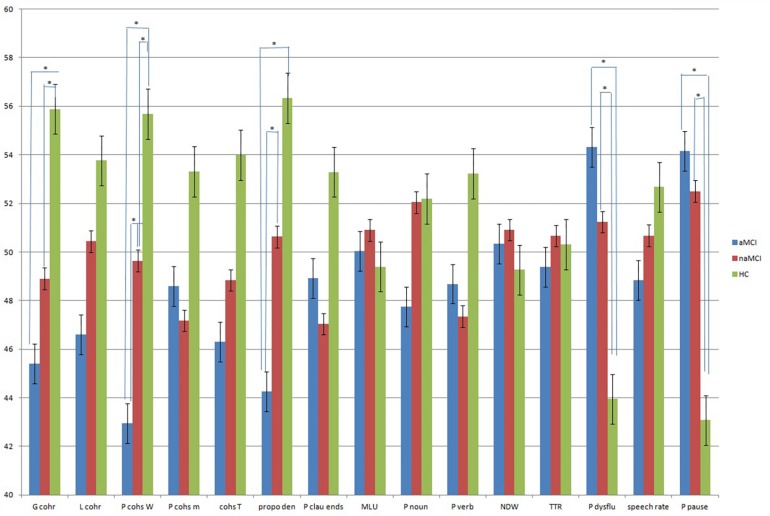
Comparison ofdiscourse measures with converted T score in aMCI, naMCI, and HC. Asterisks (^∗^) indicate discourse measures with significant group differences.

To compare the discourse performances with various measures in a unified manner, each mean value was converted to a T score (Table 4). With this converted T score, the largest group difference was in the proportion of cohesive words between the aMCI participants and the HC participants (aMCI = 42.94; HC = 55.68). Although the naMCI participants performed better in most measures compared to the aMCI participants, the discourse performance of the aMCI participants was better than that of the naMCI participants in grammaticality and fluency. Group differences and the comparison with the converted T score are presented in Figure 1.

**TABLE 4:** T score of discourse measures and group differences among aMCI, naMCI, and healthy control participants.

	**Measures**	**aMCI**	**naMCI**	**HC**	**HC-aMCI**	**HC-naMCI**	**naMCI-aMCI**
Coherence	Global coherence	45.41	48.90	55.88	**10.47**	**6.98**	3.50
	Local coherence	46.61	50.44	53.76	7.15	3.32	3.83
Cohesion	Proportion of cohesive words	42.94	49.64	55.68	**12.74**	**6.04**	**6.70**
	Proportion of cohesive morphemes	48.60	47.18	53.31	4.71	6.13	–1.42
	Cohesive ties per utterance	46.30	48.83	53.99	7.69	5.16	2.53
Proposition	Propositional density	44.26	50.63	56.33	**12.08**	5.71	**6.37**
Grammaticality	Proportion of clausal endings	48.92	47.04	53.29	4.37	6.25	–1.89
	MLU	50.04	50.90	49.39	–0.65	–1.51	0.85
Lexicality	Proportion of nouns	47.74	52.05	52.19	4.45	0.14	4.31
	Proportion of verbs	48.69	47.35	53.23	4.54	5.88	–1.34
	NDW	50.33	50.91	49.27	–1.06	–1.64	0.58
	TTR	49.37	50.67	50.31	0.94	–0.35	1.29
Fluency	Proportion of dysfluencies	54.33	51.24	43.95	**−10.38**	**−7.29**	–3.09
	Speech rate	48.85	50.67	52.67	3.83	2.00	1.82
	Proportion of pauses	54.15	52.50	43.08	**−11.07**	**−9.42**	–1.65

*aMCI, amnestic mild cognitive impairment; naMCI, non-amnestic MCI; HC, healthy control. MLU, mean length of utterances; NDW, number of different word; TTR, type token ratio. Statistically significant differences are highlighted with bold letters.*

### Discourse Measures to Differentiate aMCI Participants, naMCI Participants, and HC Participants

To determine whether the previously mentioned measures significantly differed between two groups (i.e., aMCI and naMCI, naMCI and HC, and aMCI and HC), a binary logistic regression and ROC curve analysis were performed. Firstly, to differentiate naMCI from aMCI with discourse measures, an odds ratio, a 95% confidence interval (CI), and an area under the curve (AUC) of the proportion of cohesive words and propositional density are presented in Table 5, controlling for age, education, and sex.

**TABLE 5:** Logistic regression analysis of the proportion of cohesive words and propositional density in aMCI and naMCI.

**Measures**	**aMCI (%)**	**naMCI (%)**	**OR (95% CI)**	***p***	**R^2^**	**AUC (95% CI)**
Proportion of cohesive words	22/8 (73.3%)	13/9 (59.1%)	1.342 (1.064∼1.667)	0.013	0.212	0.773 (0.643∼0.902)
Propositional density	23/7 (76.7%)	12/10 (54.5%)	1.068 (0.996∼1.146)	0.065	0.149	0.718 (0.577∼0.860)

*aMCI, amnestic mild cognitive impairment; naMCI, non-amnestic mild cognitive impairment; OR, odds ratio; CI, confidence interval; AUC, area under the curve. R^2^, Cox & Snell R^2^. Controlling for age, education, and gender; reference value: female.*

The classification table indicated that the aMCI participants were more frequently correctly classified than were the naMCI participants with both measures of the proportion of cohesive words and propositional density. Since the dependent variable in Table 5 was the naMCI participants, a 1% point increase in the proportion of cohesive words means a greater likelihood of being diagnosed with naMCI with an odds ratio of 1.342. However, the propositional density did not have the statistical ability to differentiate the naMCI participants from the aMCI participants (*p* = 0.065), although the propositional density was significantly different between the two groups.

**FIGURE 2:**
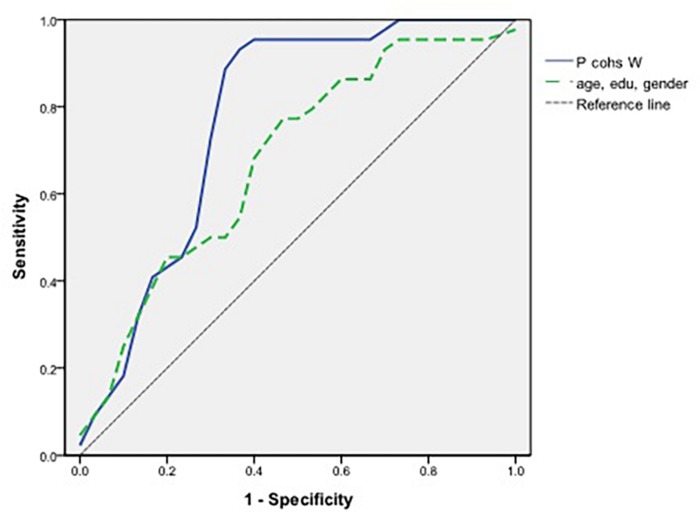
Receiver operating characteristic curve in measures of the proportion of cohesive words and controlling variables in aMCI and naMCI.

The ROC curve as a predictor of the proportion of cohesive words is presented in Figure 2. Although the AUC of the probability of controlling variables (i.e., age, years of education, and sex) was 0.680 (95% CI = 0.532–0.827), the proportion of cohesive words was sufficient as a predictor with an AUC of 0.773 (95% CI = 0.643–0.902).

Second, measures to differentiate the naMCI participants from the HC participants were analyzed in the same manner. An odds ratio, a 95% CI, and an AUC of global coherence, the proportion of cohesive words, and the proportion of dysfluencies and pauses are presented in Table 6. A score increase of one point in global coherence means a reduction of 0.027 in the likelihood of being diagnosed with naMCI. A one-point increase in the rate of the proportion of dysfluencies and pauses increased the probability of being diagnosed with naMCI with odds of 1.192 and 1.219, respectively. However, the proportion of cohesive words did not have the statistical ability to differentiate the naMCI participants from the HC group (*p* = 0.050) (Table 6).

**TABLE 6:** Logistic regression analysis of global coherence, the proportion of cohesive words, dysfluencies, and pauses in naMCI and healthy control participants.

**Measures**	**naMCI (%)**	**HC (%)**	**OR (95% CI)**	***p***	**R^2^**	**AUC (95% CI)**
Global coherence	15/7 (68.2%)	15/6 (71.4%)	0.027 (0.002∼0.445)	0.012	0.215	0.745 (0.597∼0.892)
Proportion of cohesive words	16/6 (72.7%)	14/7 (66.7%)	0.813 (0.662∼0.1000)	0.050	0.142	0.706 (0.537∼0.875)
Proportion of dysfluencies	15/7 (68.2%)	15/6 (71.4%)	1.192 (1.031∼1.378)	0.018	0.194	0.736 (0.574∼0.898)
Proportion of pauses	16/6 (72.7%)	17/4 (81.0%)	1.219 (1.064∼1.395)	0.004	0.307	0.817 (0.685∼0.949)

*naMCI, non-amnestic mild cognitive impairment; HC, healthy control; OR, odds ratio; CI, confidence interval; AUC, area under the curve. R^2^, Cox & Snell R^2^. Controlling for age, education, and gender; reference value: female.*

The AUC of the controlling variables (i.e., age, education, and sex) was 0.615 (95% CI = 0.445–0.785) in group differentiation between the naMCI participants and the HC participants. The AUC of global coherence was 0.745 (95% CI = 0.597–0.892). With the same ROC curve as the controlling variables, the AUC of the proportion of dysfluencies and pauses was 0.736 (95% CI = 0.574–0.898) and 0.817 (95% CI = 0.685–0.949), respectively. Therefore, the three measures in Table 6 were distinguishing predictors for differentiating naMCI participants from HC participants. Among the four discourse measures, the proportion of pauses had the most statistical impact in the differentiation between the naMCI group and the HC group.

Finally, an odds ratio, a 95% CI, and an AUC are presented in Table 7 to differentiate the aMCI participants and HC participants. The results indicate the likelihood of being classified in the aMCI group with a reduced discourse performance in coherence, cohesion, proposition, and fluencies. When the score of global coherence increases by one point, it reduces the odds of being diagnosed with aMCI by 0.039-fold. If the proportion of cohesive words increased by 1%, it reduced the odds of being aMCI to 0.717. In addition, per proposition and fluency measures, when the proportion of propositional density increased by 1%, it reduced the odds of being diagnosed with aMCI to 0.882. However, when the proportion of dysfluencies or pauses increased by one point, the probability of being diagnosed with aMCI increased by 1.162- and 1.280-fold, respectively (Table 7).

**TABLE 7:** Logistic regression analysis of measures in coherence, cohesion, proposition, and fluency in aMCI and healthy control participants.

**Measures**	**aMCI (%)**	**HC (%)**	**OR (95% CI)**	***p***	**R^2^**	**AUC (95% CI)**
Global coherence	23/7 (76.7%)	12/9 (57.1%)	0.039 (0.004∼0.361)	0.004	0.281	0.770 (0.643∼0.897)
Proportion of cohesive words	23/7 (76.7%)	16/5 (76.2%)	0.717 (0.588∼0.875)	0.001	0.339	0.843 (0.734∼0.952)
Propositional density	25/5 (83.3%)	16/5 (76.2%)	0.882 (0.815∼0.954)	0.002	0.325	0.821 (0.703∼0.938)
Proportion of dysfluencies	25/5 (83.3%)	14/7 (73.3%)	1.162 (1.038∼1.300)	0.009	0.238	0.752 (0.614∼0.891)
Proportion of pauses	25/5 (83.3%)	16/5 (76.2%)	1.280 (1.101∼1.488)	0.003	0.370	0.859 (0.748∼0.969)

*aMCI, amnestic mild cognitive impairment; HC, healthy control; OR, odds ratio; CI, confidence interval; AUC, area under the curve. R^2^, Cox & Snell R^2^. Controlling for age, education, and gender; reference value: female.*

The AUC of the controlling variables was 0.668 (95% CI = 0.519–0.817) in the group differentiation between the aMCI and HC groups. The AUCs of all five measures were higher than those of the controlling variables (Table 7). In conclusion, the proportion of pauses had the most statistical relevance for the group differentiation between the aMCI participants and the HC participants with an AUC of 0.859. The second measure among the five measures with a higher AUC than that of the controlling variables was the proportion of cohesive words, with an AUC of 0.843.

### Statistical Comparison of Discourse Measures

Three measures could differentiate the naMCI participants from the HC participants and five measures could distinguish between the aMCI participants and the HC participants. However, to explain the dependent variables (i.e., participants being diagnosed as naMCI or aMCI) with the minimum number of predictors, a binary multiple logistic regression analysis with the previously mentioned three or five measures was simultaneously performed.

For differentiating between naMCI participants and HC participants, two measures of the proportion of pauses and global coherence were statistically selected in this order with the forward method when three measures were initially entered. An odds ratio, a 95% CI, and an AUC are presented in Table 8, and comparisons to those of the logistic regression analysis were conducted separately for each measure.

**TABLE 8:** Logistic regression results for comparison of discourse measures to differentiate naMCI from healthy control participants.

	**Forward (conditional)**			**Separate**		
**Discourse measures**	**OR (95% CI)**	***R*^2^**	**AUC (95% CI)**	**OR (95% CI)**	**R^2^**	**AUC (95% CI)**
Proportion of pauses	1.224^∗^ (1.053∼1.424)			1.219^∗^ (1.064∼1.395)	0.215	0.817 (0.685∼0.949)
Global coherence	0.033^∗^ (0.002∼0.717)			0.027^∗^ (0.002∼0.445)	0.307	0.745 (0.597∼0.892)
These		0.367	0.868 (0.761∼0.975)	–	–	–

*^∗^*p* < 0.05. naMCI, non-amnestic mild cognitive impairment; OR, odds ratio; CI, confidence interval; AUC, area under the curve. Forward (conditional), entered four measures of the proportion of pauses, dysfluencies, and cohesive words, and global coherence with the forward conditional method; Separate, conducted separately for each measure. These, all two measures. R^2^, Cox & Snell R^2^.*

**FIGURE 3:**
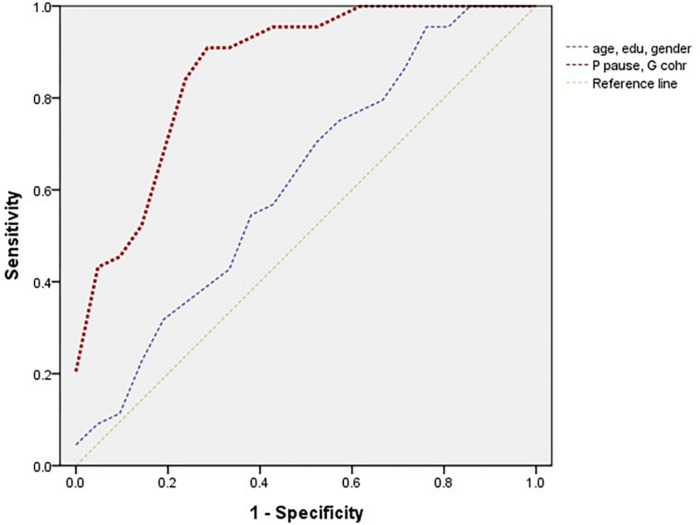
Receiver operating characteristic curve in two influential measures (i.e., the proportion of pauses and global coherence) and controlling variables in naMCI and HC.

Both the AUC and regression results using all three measures proved that the proportion of pauses was the most influential among them. In addition, the AUC of these selected two measures had a value of 0.868. Although the AUC of the controlling variables was 0.615 (95% CI = 0.445–0.785), the two measures together were efficient for group differentiation. The ROCs of these three measures and controlling variables are presented in Figure 3.

**FIGURE 4:**
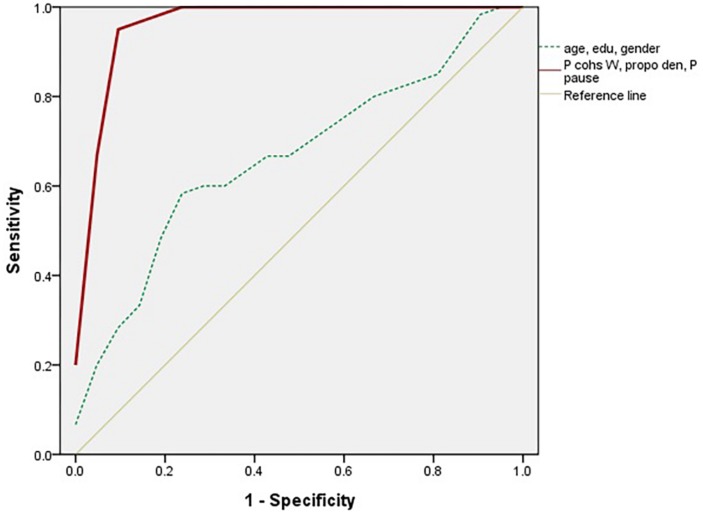
Receiver operating characteristic curve in three influential measures (i.e., the proportion of cohesive words and pauses and propositional density) and controlling variables in aMCI and HC.

For differentiating aMCI from the HC, the five measures (i.e., global coherence, the proportion of cohesive words, propositional density, the proportion of dysfluencies and pauses) were entered. The proportion of cohesive words and pauses and propositional density were the influential measures for discerning the two groups (Table 9). The ROC curves of the three measures and controlling variables are presented in Figure 4.

**TABLE 9:** Logistic regression results for comparison of discourse measures to differentiate aMCI from healthy control participants.

	**Forward (conditional)**			**Separate**		
**Discourse measures**	**OR (95% CI)**	**R^2^**	**AUC (95% CI)**	**OR (95% CI)**	**R^2^**	**AUC (95% CI)**
Proportion of cohesive words	0.715^∗^ (0.544∼0.940)			0.717 (0.588∼0.875)	0.339	0.843 (0.734∼0.952)
Propositional density	0.839^∗^ (0.722∼0.974)			0.882 (0.815∼0.954)	0.325	0.821 (0.703∼0.938)
Proportion of pauses	1.371^∗^ (1.074∼1.751)			1.280 (1.101∼1.488)	0.370	0.859 (0.748∼0.969)
These		0.584	0.965 (0.000∼1.000)		–	–

*^∗^*p* < 0.05. aMCI, amnestic mild cognitive impairment; OR, odds ratio; CI, confidence interval; AUC, area under the curve. Forward (conditional), entered four measures of the proportion of cohesive words, propositional density, and the proportion of dysfluencies and pauses with the forward conditional method; separate, conducted separately for each measure. These, all three measures. R^2^, Cox & Snell R^2^.*

### Correlation Between Discourse Measures and Cognitive Functions in MCI

To investigate the cognitive functions that are associated with discourse measures, the measures and the scores of the neuropsychological cognitive test of 30 aMCI and 22 naMCI participants were examined using Pearson correlation analysis (Tables 10, 11). The following results of the correlation analysis are described per discourse measures that had significant group differences.

**TABLE 10:** Correlation between discourse measures and cognitive functions in aMCI.

				**Proportion**	**Proportion**	**Cohesive**		**Proportion**								
		**Global**	**Local**	**of cohesive**	**of cohesive**	**ties per**	**Propositional**	**of clausal**		**Proportion**	**Proportion**			**Proportion of**	**Speech**	**Proportion**
		**coherence**	**coherence**	**words**	**morphemes**	**utterance**	**density**	**endings**	**MLU**	**of nouns**	**of verbs**	**NDW**	**TTR**	**dysfluencies**	**rate**	**of pauses**
Attention	Digit span test															
	Forward	0.227	0.209	**0.439^∗^**	0.150	0.304	0.135	0.026	0.065	0.049	–0.186	0.145	–0.097	–0.047	0.194	–0.158
	Backward	0.098	–0.005	–0.027	–0.017	0.075	–0.084	0.147	0.064	–0.204	0.084	0.063	–0.225	0.038	–0.044	0.125
Language	Confrontation naming															
	K-BNT-15	0.329	**0.476^∗∗^**	0.122	–0.112	–0.235	**0.473^∗∗^**	–0.298	**−0.480^∗∗^**	**0.569^∗∗^**	–0.028	–0.148	0.204	0.025	0.019	0.092
Visuospatial function	Rey complex figure test															
	Score	0.042	0.202	0.078	–0.133	0.052	0.028	–0.085	0.078	0.305	–0.030	0.140	0.195	–0.176	0.055	0.017
	Time	–0.237	–0.350	–0.040	–0.063	–0.194	–0.242	–0.087	–0.169	–0.171	0.033	–0.066	–0.138	0.189	–0.163	0.032
Memory	Seoul verbal learning test															
	Immediate recall	**0.550^∗∗^**	0.349	0.263	0.030	–0.012	0.299	–0.244	–0.159	**0.362^∗^**	–0.049	–0.226	0.305	0.107	–0.031	0.095
	Delayed recall	**0.378^∗^**	**0.504^∗∗^**	–0.020	0.000	0.085	**0.377^∗^**	0.193	0.183	**0.371^∗^**	–0.193	–0.116	0.173	0.302	–0.042	–0.050
	Rey complex figure test															
	Immediate recall	0.145	**0.376^∗^**	–0.128	–0.034	–0.040	0.326	–0.077	–0.175	**0.374^∗^**	–0.017	0.152	0.033	0.178	0.055	–0.054
	Delayed recall	0.107	**0.375^∗^**	–0.155	–0.163	–0.102	0.275	–0.046	–0.188	**0.408^∗^**	–0.139	0.151	0.030	0.161	0.050	–0.073
Executive Function	Verbal fluency															
	Semantic	**0.553^∗∗^**	**0.514^∗∗^**	0.023	–0.008	–0.034	**0.517^∗∗^**	**−0.391^∗^**	–0.098	**0.675^∗∗^**	0.051	–0.101	**0.480^∗∗^**	–0.057	0.053	–0.026
	Phonemic	**0.402^∗^**	**0.387^∗^**	–0.206	0.226	0.360	0.109	0.154	0.284	0.000	–0.334	0.308	0.008	0.133	0.252	–0.208
	Color word stroop test															
	Word reading															
	Correct response	–0.009	–0.072	0.050	0.156	0.048	–0.121	0.001	–0.013	0.011	**0.365^∗^**	0.156	0.126	–0.096	–0.022	0.207
	Time	**−0.482^∗∗^**	**−0.411^∗^**	–0.227	–0.252	–0.147	–0.169	0.033	0.114	–0.145	0.089	–0.097	–0.159	0.122	**−0.372^∗^**	0.210
	Time per item	**−0.380^∗^**	–0.296	–0.193	–0.263	–0.135	–0.089	0.029	0.106	–0.118	–0.051	–0.141	–0.164	0.134	–0.290	0.101
	Color reading															
	Correct response	0.127	0.199	0.247	0.072	0.040	0.124	–0.098	–0.095	0.268	0.144	–0.120	0.229	–0.151	0.089	–0.005
	Time	–0.258	–0.211	0.112	0.223	0.197	–0.078	0.203	–0.034	–0.106	0.062	0.060	–0.197	0.043	–0.005	–0.091
	Time per item	0.190	0.214	–0.153	–0.269	–0.185	0.114	–0.134	0.113	0.068	–0.131	–0.106	0.149	0.024	–0.012	0.053

*Values are correlation coefficients. ^∗^*p* < 0.05 and ^∗∗^*p* < 0.01 are specified in bolded terms. MLU, mean length of utterances; NDW, number of different words; TTR, type token ratio. K-BNT-15; the 15-item Korean version of the Boston Naming Test; Semantic verbal fluency, the mean score of two semantic verbal fluencies (animal and supermarket); phonemic verbal fluency: the mean score of three phonemic verbal fluencies (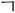, 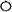, 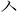).*

**TABLE 11:** Correlation between discourse measures and cognitive functions in naMCI.

				**Proportion**	**Proportion**	**Cohesive**		**Proportion**								
		**Global**	**Local**	**of cohesive**	**of cohesive**	**ties per**	**Propositional**	**of clausal**		**Proportion**	**Proportion**			**Proportion of**	**Speech**	**Proportion**
		**coherence**	**coherence**	**words**	**morphemes**	**utterance**	**density**	**endings**	**MLU**	**of nouns**	**of verbs**	**NDW**	**TTR**	**dysfluencies**	**rate**	**of pauses**
Attention	Digit span test															
	Forward	0.177	**0.445^∗^**	0.019	0.026	–0.048	0.320	–0.049	–0.038	0.056	0.053	0.030	0.138	–0.349	–0.153	–0.021
	Backward	0.102	0.185	–0.183	–0.374	–0.178	0.212	–0.359	0.302	0.404	–0.209	0.110	0.372	–0.131	0.142	–0.133
Language	Confrontation naming test															
	K-BNT-15	0.131	–0.025	0.260	–0.390	–336	**0.432^∗^**	–0.090	–0.304	0.281	0.049	–0.073	0.059	0.125	–0.254	0.050
Visuospatial function	Rey complex figure test															
	Score	0.061	0.092	0.259	–0.264	–0.009	0.227	–0.032	0.270	0.023	–0.044	0.243	–0.010	–0.342	0.286	**−0.484^∗^**
	Time	–0.031	–0.029	–0.321	0.281	–117	–0.270	–0.006	0.006	–0.084	0.022	0.039	0.010	–0.041	0.062	–0.018
Memory	Seoul verbal learning test															
	Immediate recall	0.255	0.103	–0.071	–0.130	0.132	0.399	–0.038	**0.441^∗^**	–0.056	**0.424^∗^**	–0.189	0.205	–0.176	–0.087	0.081
	Delayed recall	0.233	0.036	0.075	–0.179	0.106	0.354	–0.051	0.301	0.314	0.328	–0.256	0.292	–0.027	–0.031	0.229
	Rey complex figure test															
	Immediate recall	0.115	0.374	**0.458^∗^**	–0.172	–0.003	0.114	0.137	0.146	0.025	–0.193	0.012	–0.080	–0.058	0.112	–0.140
	Delayed recall	0.123	0.291	**0.469^∗^**	–0.036	0.020	0.124	0.233	0.077	–0.154	0.027	–0.030	–0.161	–0.185	0.246	–0.241
Executive Function	Verbal fluency															
	Semantic	–0.081	0.113	0.163	–0.255	0.063	0.258	–0.162	0.325	0.239	–0.172	0.265	0.200	–0.015	–0.056	–0.031
	Phonemic	0.284	0.237	–0.087	–0.312	–0.104	0.303	–0.089	0.362	0.056	0.074	–0.031	0.211	–0.178	0.115	–0.158
	Color Word Stroop Test															
	Word reading															
	correct response	–0.256	–0.211	**0.428^∗^**	–0.200	0.135	–0.011	–0.210	0.195	–0.269	–0.078	0.065	–0.098	–0.342	0.303	–0.285
	time	0.266	0.187	–0.319	0.277	–0.008	–0.147	0.230	–0.107	–0.106	0.207	–0.278	0.032	0.215	–0.241	0.226
	time per item	0.279	0.203	–0.345	0.273	–0.025	–0.130	0.230	–0.115	–0.072	0.202	–0.262	0.043	0.228	–0.253	0.229
	Color reading															
	correct response	–0.106	–0.061	0.278	–0.074	–0.074	0.142	–0.015	0.311	0.255	0.117	–0.057	0.198	–0.022	0.011	0.186
	time	0.150	0.150	**−0.661^∗∗∗^**	0.227	0.227	–0.084	0.004	–0.030	–0.243	0.405	–0.024	–0.043	–0.011	–0.392	0.248
	time per item	0.147	0.067	–0.286	0.116	0.116	–0.112	0.027	–0.283	–0.299	–0.010	0.033	–0.236	–0.081	0.028	–0.150

*Values are correlation coefficients. ^∗^*p* < 0.05 and ^∗∗^*p* < 0.01 are specified in bolded terms. MLU, mean length of utterances; NDW, number of different words; TTR, type token ratio. K-BNT-15; the 15-item Korean version of the Boston Naming Test; semantic verbal fluency: the mean score of two semantic verbal fluencies (animal and supermarket); phonemic verbal fluency: the mean score of three phonemic verbal fluencies (??, ??, ??).*

#### Discourse Measures and Cognition in aMCI

Regarding measures of coherence, global coherence was positively associated with the SVLT immediate recall and delayed recall (*r* = 0.550, *p* < 0.002; *r* = 0.378, *p* < 0.039) and positively with semantic and phonemic verbal fluencies (*r* = 0.553, *p* < 0.002; *r* = 0.402, *p* < 0.028), and negatively with the word reading time and the time per item of the CWST (*r* = −0.482, *p* < 0.007; *r* = −0.380, *p* < 0.038). The proportion of cohesive words was also positively associated with the Digit Span Test forward (*r* = 0.439, *p* < 0.015). Propositional density was positively correlated with the K-BNT-15 (*r* = 0.473, *p* < 0.008), delayed recall of the SVLT (*r* = 0.377, *p* < 0.040), and semantic verbal fluency (*r* = 0.517, *p* < 0.003) (Table 10).

#### Discourse Measures and Cognition in naMCI

The proportion of cohesive words were positively correlated with immediate recall and delayed recall in the RCFT (*r* = 0.458, *p* < 0.032; *r* = 0.469, *p* < 0.028), and word reading correct responses in the CWST (*r* = 0.428, *p* < 0.047). It was negatively associated with the color reading time in the CWST (*r* = −0.661, *p* < 0.001). Propositional density was positively related only to the K-BNT-15 (*r* = 0.432, *p* < 0.044). The proportion of pauses in the naMCI participants was negatively associated with the RCFT score (*r* = −0.484, *p* < 0.022) (Table 11).

## Discussion

The primary goal of this study was to identify the differential discourse measures of MCI participants according to the MCI subtypes without being limited to discourse genres or measurements. The discourse performance of the aMCI participants, naMCI participants, and HC participants revealed that global coherence, cohesion, propositional density, and measures of fluency could distinguish language performance among the three groups. Of note, the aMCI and naMCI participants differed in cohesion and propositions. In addition, cohesion and pauses of MCI discourse were the most distinguishable from discourse production in the HC participants. Various cognitive functions were involved in the previously mentioned measures.

### Coherence in MCI

Global coherence (i.e., topic maintenance) was lower in the MCI groups than in the HC group. aMCI and naMCI participants experience difficulty in processing conceptual organizational levels of discourse production (Sherratt, 2007; Joanette and Brownell, 2012). The MCI participants yielded more repetitions and egocentric information than did the HC participants. The results of global coherence in the aMCI participants in this study are partially consistent with those of a previous study (Drummond et al., 2015), because the macropropositions in the aMCI group were preserved and irrelevant micropropositions were more frequent in the aMCI group than in the HC group. The aMCI participants might experience slight difficulty in top-down planning and retrieval of episodic memories (Drummond et al., 2015). Our results partially support the relatively preserved ability of episodic memory as only 10.03% of the aMCI participants and 8.18% of the naMCI participants scored 1 point on global coherence for utterances from our narrative task of “raising children.” Thus, MCI participants could at least retrieve topic-relevant episodic memories. However, most utterances in the MCI group were scored as three or two points. For example, difficulties in the topic shifting of MCI discourse were noticed, in that the topic shift seldom happened from strenuous, egocentric experiences in life (i.e., two scored utterances) to difficult experiences about raising children during the narrative task, and from personal travel episodes (i.e., two scored utterances) to planning and preparing a trip in the “Trip to Jeju Island” task, which might have been caused by insufficient suppression of irrelevant information that resulted in persistence with an inadequate substructure (Gernsbacher, 1990).

In addition, the aMCI group of this study included 20 multiple-domain aMCI participants with reduced executive function. The correlation between memory, executive functions, and global coherence measures in the aMCI participants demonstrated that both memory and execution are involved in the maintenance of the topic. The verbal learning task used in this study requires retaining, encoding, and retrieval of the correct information. Additionally, this task requires the conscious formulation of word groups, planning, and organizing information, which indicates cognitive flexibility (Le et al., 2014). These processes are also imperative during discourse production, in that a narrative that is consistent with the topic can be produced when information regarding the narrative is correctly formulated, planned, and organized with the designated topic in mind. In addition, the correlation between verbal fluency and global coherence revealed that processing speed and inhibition are needed to maintain the theme of discourse as supported by the results of a study that reported that verbal fluency tasks are related to executive functions of processing speed and inhibition (McDowd et al., 2011). Thus, producing a coherent discourse while maintaining a topic and switching between subtopics requires the ability to search for words based on semantic knowledge and to suppress repetitive or irrelevant information that is off topic (Lee and Kim, 2019).

However, there was no correlation between cognitive function and the global coherence measure in the naMCI participants. Per our demographic information, only 50% of the naMCI participants had impaired executive function compared to two-thirds of the aMCI participants. The lower global coherence measure scores in the naMCI participants than in the HC participants may be due to repetition of questions or utterances due to difficulty in initiating the production of discourse.

### Cohesion in MCI and Its Subtypes

A notable finding of this study was that there were significant differences among all three groups in the proportion of cohesive words. This measure was also the most significant measure for discerning the MCI participants from the HC participants. The number of cohesive words in this study was calculated by the sum of the references, exact repetitions of words or phrases, synonymous phrases, superordinate designates, subordinate exemplars, and conjunctions (Glosser and Deser, 1991). Among these six cohesive measures, the reference type (i.e., demonstratives, personal pronouns, and determiners) amounted to the most frequently used cohesive word and was the most differential among the three groups. Particularly, personal pronouns that designated the first person or children in a narrative and demonstrative pronoun for things, directions, and places that were included in specific episodes were most frequently used during the narrative task. Demonstrative pronouns connect the previously mentioned words or phrases with the other meanings of sequential utterances. For example, an episode of a son being sick in the narrative task of raising children could be referred to as “this” and meanings of sequential utterances can be extended such as “this made me sad” or “this made me a fool.” The difficulties in using cohesive words of this reference type in discourse production have an effect in developing networks of meanings across utterances, because this reference connects lower and higher levels of discourse (Ellis et al., 2005; Joanette and Brownell, 2012).

Regarding semantic-lexical abilities, the MCI participants exhibited a higher proportion of cohesive words in the narrative task of “raising children” than in the other two tasks. In contrast, the HC participants exhibited the highest proportion of cohesive words in the “Trip to Jeju Island” task among all three tasks. The lower cohesion on the “Trip to Jeju Island” task performed for the aMCI and the naMCI participants resulted from the less frequently used subordinate exemplars. Particularly, “preparation materials” contained subordinate words such as “toiletries,” “clothes,” and “snacks (food)”; likewise, a superordinate word of “sightseeing” was followed by specific places and activities such as “beaches” or “museums.” However, the MCI participants could not generate relevant open lexical words sufficiently in the same task. Participants with MCI may not form lexical chains at an intersentential level to the same degree that the HC participants can (Mentis and Prutting, 1987). Thus, the difference between the MCI participants and the HC participants in cohesion may result from debilitating semantic-lexical abilities at the sentential level in those with MCI.

Concerning the cognitive functions that are associated with cohesion, the score of the Digit Span Test forward and the proportion of cohesive words were related in the aMCI participants. Attentional capacity in discourse plays an important role in generating words, particularly in noun use (March et al., 2009). In this study, higher attention was associated with better performance in cohesion in the aMCI participants.

According to the correlation results in the naMCI participants, immediate recall and delayed recall in the RCFT were associated with the proportion of cohesive words. Recall tests in the RCFT assess not only non-verbal memory (visual memory) but also cognitive abilities of planning, organization, and problem solving (Noggle and Dean, 2012). Cohesion in the naMCI participants was the lowest in the “Trip to Jeju Island” task among the three tasks, as it requires planning and problem solving with cognitive flexibility (Fleming and Harris, 2008). The time of color reading in the CWST was also negatively associated with cohesion in the naMCI participants. The slow cognitive processing speed of the naMCI participants might prevent the proper use of cohesive words during the production of discourse.

### Propositional Density in MCI and Its Subtypes

Among the three groups, only the aMCI participants exhibited lower density of propositions; the naMCI participants and the HC participants exhibited >70% propositional density. This result is consistent with that of a previous study, as aMCI participants generated more irrelevant micropropositions than did the normal control group (Drummond et al., 2015). The semantic-pragmatic impairment might influence the generation of irrelevant micropropositions (Drummond et al., 2015).

Discourse of the aMCI participants had less density than did those of the naMCI participants, which was consistent with the hypothesis of this study. The discourse in the aMCI group was not efficient due to unnecessary redundancies and fillers, and such discourse contained more completely off-topic propositions than did the naMCI participants’ discourse. During discourse production, the aMCI participants may have strategies or symptoms of using repetitive propositions for maintaining their speech continuously and compensating for their discourse discontinuity resulting from retrieval difficulties of appropriate words or information due to impaired memory.

In the aMCI group, producing discourse with a higher propositional density requires not only lexical retrieval ability, but also better memory and executive function, according to the correlation analysis. Since the aMCI participants were composed of 26 multiple domain aMCI participants including 20 participants with reduced execution and 12 participants with reduced language ability, reduced abilities of memory with other cognitive functions such as executions and language skills might additively affect the efficiencies and informativeness of their discourse performances.

The correlation between propositional density and verbal fluency indicated that a lower propositional density resulted from a decreased ability to initiate utterances spontaneously, which is associated with processing speed (McDowd et al., 2011), and to maintain the generation of discourse, which is related to inhibitory and switching ability (McDowd et al., 2011; Lee and Kim, 2019). In this study, the aMCI participants also frequently repeated task questions when they began to speak or rephrased predicates during their discourse production due to difficulties in formulating utterances or word retrievals, which lowered their propositional density.

### Fluency in MCI

The proportions of dysfluencies and pauses were higher in both the aMCI participants and the naMCI participants than in the HC participants; however, there was no difference between the two subtypes of MCI. Dysfluency in this study was the aggregate value of the frequencies of fillers, pauses, repetitions, and revisions and this result is consistent with previous findings of dysfluent words (Harris et al., 2008; Fleming, 2014) in MCI and fluency factors (Mueller et al., 2018) in subclinical MCI. The difference in the proportion of dysfluencies between the MCI participants and the HC participants reflects subtle changes in communicative effectiveness that cannot be detected in noun or verb naming tests (Harris et al., 2008). Dysfluencies also serve as a coordinative function to gain more time for access to certain information or words in order to decrease word reformulations and substitution errors (Schmitter-Edgecombe et al., 2000).

Both MCI groups exhibited a higher proportion of pauses in their total speaking time than did the HC participants. In particular, pauses were proven to be the most differential discourse measure among various discourse measures for comparing MCI participant’s discourse with those of the HC participants. Since there is no research focused on MCI pauses in discourse, we speculate that longer pauses occur in MCI participants due to reduced linguistic and cognitive processing speeds during discourse production. Although pauses in the aMCI participants were not correlated with cognitive function, visuospatial function in the RCFT in the naMCI participants was positively correlated with the proportion of pauses. Initiating and processing discourse production might require both visuospatial ability (i.e., in the picture description of this study) and also planning, organization, and problem-solving (Noggle and Dean, 2012).

In this study, an interesting tendency was observed in the three groups regarding the initiating time of the “crosswalk” picture task. Participants who experienced a delay of >2 s at the beginning were composed of 13 naMCI participants (59.1%), 16 aMCI participants (53.3%), and six HC participants (20%). The measurement of the response time, along with the common word retrieval tests, can be useful to differentiate those with MCI from the HC participants (Taler and Phillips, 2008). The initiation time of the “crosswalk” picture task may support this claim. According to a previous study that measured the proportion of correct responses and the switching of verbal fluency according to MCI subtypes, the aMCI participants and the HC participants generated more words and exhibited more switching over to the previous 30 s of the 60 s period, but the naMCI participants generated the fewest words and less switching during the interval of 30 s (Weakley et al., 2013). The naMCI group might require more time for initiation of word retrieval, or they might have different symptoms (i.e., delayed initiation) or strategic processes compared to the aMCI participants and the HC participants in order to initiate discourse production.

### Limitations

This study has several limitations. First, cognitive functions among the three groups were not compared, because different cognitive tests were performed on the MCI participants and HC participants. Further research warrants comparing cognitive functions with discourse performances between the three groups. Second, the MCI groups were not divided using cognitive domain-specific classifications such as single domain and multiple domains. Further research could compare the discourse performances per domain-specific classifications of MCI participants to determine how the cognitive abilities of MCI affect discourse performance. Third, this study was not counterbalanced in the practice of discourse tasks. Although there was a picture description task between the narrative task of “raising children” and the “Trip to Jeju Island” task, there were a few participants who voiced utterances regarding their children during the third task. When using a variety of discourse tasks, the tasks should be counterbalanced across participants.

## Conclusion

The conflicting conclusions regarding the language impairments of MCI participants have been broadly discussed with standardized and non-standardized language tests. This study intended to delineate the language-specific impairments of MCI participants. Through the frame of a multi-layered model of discourse processing and various measures and tasks that have been reported in previous studies, this study verified several hypotheses regarding discourse performance in MCI.

Per the subtypes of MCI, the discourse of the aMCI participants were less efficient and less cohesive than those of the naMCI participants due to their reduced semantic memory and execution. In addition, the naMCI participants were less coherent and cohesive and produced longer pauses during discourse production than did the HC participants due to their reduced attentional and executive functions. The discourse of aMCI participants were less coherent, cohesive, and efficient, and produced more dysfluencies and longer pauses than did those of the HC participants due to their impaired memory and frontal/executive functions, as was predicted.

The frequencies of cohesive words and pauses, to compensate for reduced cognitive function, such as semantic memory and execution, could be presented through the findings here. Considering these reports, clinicians will be able to assess language impairments as per the subtypes of aMCI and naMCI, and as per the HCs. Using this discourse analysis for examining language impairments in individuals with MCI will characterize and generalize detailed reductions in the language skills of those with MCI. In addition, the methods that were used in this study to assess coherence, cohesion, proposition and pauses and distinguish among the three groups can be used in early detection of dementia. On the basis of the methods of this study, further studies may design a scoring system for the performance of discourse for people with early onset dementia or neurological diseases. Therefore, measurement of discourse performance can then be considered as a clinical diagnostic criterion to reflect cognitive decline in individuals with early onset dementia.

## Ethics Statement

This study was carried out in accordance with the recommendations of the “Severance Hospital Yonsei University’s Institutional Review Board” and “Kangbuk Samsung Hospital’s Institutional Review Board.” All subjects gave written informed consent in accordance with the Declaration of Helsinki.

## Author Contributions

BK designed the study, conducted the statistical analysis, and drafted the manuscript. HK revised the study, and provided the critical and intellectual content for the study. YK helped to collect the data and conduct the experiments.

## Conflict of Interest Statement

The authors declare that the research was conducted in the absence of any commercial or financial relationships that could be construed as a potential conflict of interest.
