# Supplementary material for: Discourse Measures to Differentiate Between Mild Cognitive Impairment and Healthy Aging
Source: Front Aging Neurosci. 2019 Aug 21;11:221. doi: 10.3389/fnagi.2019.00221 (PMC6714864; doi:10.3389/fnagi.2019.00221)
Supplement: Supplementary file 1 [file Data_Sheet_1.docx]

Supplementary Material

Discourse Measures to Differentiate Between Mild Cognitive Impairment and Normal Aging

Bo Seon Kim, Yong Bum Kim, HyangHee Kim*

*** Correspondence:** HyangHee Kim: [h.kim@yonsei.ac.kr](mailto:h.kim@yonsei.ac.kr)

# Supplementary Tables

**Table 1** Demographic data for aMCI patients

| Patients | Age | Gender | Domains of Cognitive Impairment | Education  (years) | K-MMSE | GDS |
| --- | --- | --- | --- | --- | --- | --- |
| 1 | 72 | F | Mem | 6 | 29 | 11 |
| 2 | 76 | M | Mem | 14 | 14 | 3 |
| 3 | 62 | F | Mem | 9 | 9 | 7 |
| 4 | 62 | F | Mem | 10 | 10 | 2 |
| 5 | 78 | F | Mem, Lang | 12 | 29 | 9 |
| 6 | 80 | F | Mem, Lang | 12 | 26 | 5 |
| 7 | 80 | F | Mem, Lang | 10 | 25 | 7 |
| 8 | 60 | M | Mem, Lang | 12 | 28 | 9 |
| 9 | 75 | M | Mem, Lang | 6 | 24 | 14 |
| 10 | 79 | F | Mem, Exe | 6 | 23 | 10 |
| 11 | 83 | M | Mem, Exe | 16 | 28 | 3 |
| 12 | 83 | F | Mem, Exe | 6 | 23 | 13 |
| 13 | 69 | F | Mem, Exe | 6 | 27 | 6 |
| 14 | 67 | F | Mem, Visuo | 6 | 29 | 13 |
| 15 | 75 | F | Mem, Exe, Visuo | 16 | 27 | 4 |
| 16 | 77 | M | Mem, Exe, Visuo | 12 | 26 | 9 |
| 17 | 78 | M | Mem, Exe, Visuo | 12 | 23 | 4 |
| 18 | 76 | F | Mem, Exe, Visuo | 6 | 29 | 10 |
| 19 | 67 | F | Mem, Exe, Visuo | 9 | 27 | 3 |
| 20 | 76 | F | Mem, Exe, Visuo | 12 | 25 | 3 |
| 21 | 66 | M | Mem, Exe, Visuo | 9 | 27 | 13 |
| 22 | 71 | F | Mem, Exe, Lang | 6 | 27 | 1 |
| 23 | 69 | M | Mem, Exe, Att | 9 | 26 | 12 |
| 24 | 83 | M | Mem, Exe, Visuo, Lang | 16 | 25 | 8 |
| 25 | 83 | F | Mem, Exe, Visuo, Lang | 16 | 28 | 6 |
| 26 | 74 | M | Mem, Exe, Visuo, Lang | 16 | 25 | 2 |
| 27 | 74 | M | Mem, Exe, Visuo, Lang | 16 | 23 | 1 |
| 28 | 74 | F | Mem, Exe, Visuo, Lang | 6 | 25 | 5 |
| 29 | 74 | F | Mem, Exe, Visuo, Lang, Att | 9 | 26 | 6 |
| 30 | 71 | F | Mem, Exe, Visuo, Lang, Att | 12 | 28 | 8 |
| Range | 60~83 |  |  | 6~16 | 23~29 | 1~14 |
| Mean | 73.80 |  |  | 10.40 | 26.63 | 6.90 |
| SD | 6.41 |  |  | 3.77 | 2.19 | 3.92 |

aMCI: amnestic mild cognitive impairment; K-MMSE: the Korean version of the Mini-Mental State Examination; GDS: the 15 item short version of Geriatric Depression Scale; Mem: memory; Lang: language; Exe: execution; Visuo: visuospatial function; Att: attention

Cognitive functions are considered as being impaired with Z score below -1SD of test scores from each domain.

**Table 2** Demographic data for naMCI patients

| Patients | Age | Gender | Domains of Cognitive Impairment | Education  (years) | K-MMSE | GDS |
| --- | --- | --- | --- | --- | --- | --- |
| 1 | 75 | F | Execution | 9 | 28 | 5 |
| 2 | 79 | F | Execution | 6 | 28 | 6 |
| 3 | 68 | F | Execution | 6 | 26 | 2 |
| 4 | 75 | F | Execution | 6 | 27 | 3 |
| 5 | 73 | F | Execution | 9 | 28 | 1 |
| 6 | 74 | M | Execution | 16 | 29 | 1 |
| 7 | 74 | F | Execution | 6 | 27 | 5 |
| 8 | 73 | M | Visuospatial function | 9 | 29 | 3 |
| 9 | 62 | F | Visuospatial function | 6 | 26 | 7 |
| 10 | 74 | F | Visuospatial function | 6 | 29 | 10 |
| 11 | 76 | M | Visuospatial function | 12 | 29 | 7 |
| 12 | 72 | F | Visuospatial function | 16 | 29 | 2 |
| 13 | 61 | M | Language | 16 | 28 | 2 |
| 14 | 64 | M | Language | 12 | 29 | 11 |
| 15 | 66 | F | Language | 6 | 26 | 6 |
| 16 | 65 | F | Language | 6 | 27 | 3 |
| 17 | 63 | F | Attention | 12 | 29 | 3 |
| 18 | 62 | F | Attention | 6 | 29 | 8 |
| 19 | 84 | M | Execution, Visuospatial function | 16 | 25 | 1 |
| 20 | 68 | F | Execution, Visuospatial function | 12 | 30 | 7 |
| 21 | 71 | F | Execution, Visuospatial function | 11 | 23 | 14 |
| 22 | 63 | F | Execution, Attention | 6 | 23 | 13 |
| Range | 61~84 |  |  | 6~16 | 23~30 | 1~14 |
| Mean | 70.09 |  |  | 9.55 | 27.45 | 5.50 |
| SD | 6.27 |  |  | 3.90 | 1.95 | 3.28 |

naMCI: non-amnestic mild cognitive impairment; K-MMSE: the Korean version of the Mini-Mental State Examination; GDS: the 15 item short version of Geriatric Depression Scale

Cognitive functions are considered as being impaired with Z score below -1SD of test scores from each domain.

**Table 3** Demographic data for the HC

| Patients | Age | Gender | Education  (years) | K-MMSE | GDS |
| --- | --- | --- | --- | --- | --- |
| 1 | 69 | F | 6 | 27 | 12 |
| 2 | 79 | F | 6 | 27 | 3 |
| 3 | 80 | F | 12 | 29 | 3 |
| 4 | 71 | F | 11 | 26 | 2 |
| 5 | 76 | F | 6 | 28 | 7 |
| 6 | 64 | F | 9 | 27 | 3 |
| 7 | 64 | F | 9 | 26 | 9 |
| 8 | 67 | F | 9 | 28 | 2 |
| 9 | 67 | F | 12 | 28 | 2 |
| 10 | 64 | F | 9 | 27 | 6 |
| 11 | 81 | F | 6 | 26 | 3 |
| 12 | 77 | F | 11 | 26 | 6 |
| 13 | 71 | F | 6 | 26 | 4 |
| 14 | 75 | F | 6 | 28 | 5 |
| 15 | 65 | M | 12 | 27 | 1 |
| 16 | 64 | F | 6 | 27 | 4 |
| 17 | 66 | F | 6 | 28 | 2 |
| 18 | 87 | F | 6 | 26 | 4 |
| 19 | 71 | M | 9 | 27 | 1 |
| 20 | 72 | M | 16 | 29 | 4 |
| 21 | 80 | F | 6 | 28 | 7 |
| Range | 64~87 |  | 6~16 | 26~29 | 1~12 |
| Mean | 71.90 |  | 8.52 | 27.19 | 4.29 |
| SD | 6.84 |  | 2.91 | 0.98 | 2.76 |

HC: healthy control; K-MMSE: the Korean version of the Mini-Mental State Examination; GDS: the 15 item short version of Geriatric Depression Scale

# Supplementary Figures

**Figure 1** Scatter plot of global coherence scores in aMCI, naMCI, and HC

aMCI: amnestic mild cognitive impairment; naMCI: non-amnestic mild cognitive impairment; HC: healthy control

The y-axis is global coherence scores (range: 1.82~3.48) of each participant; the x-axis indicates the individual numbers of aMCI patients (1~30), naMCI patients (1~22), and the HC (1~21).

**Figure 2** Scatter plot of the proportion of cohesive words in aMCI, naMCI, and HC

aMCI: amnestic mild cognitive impairment; naMCI: non-amnestic mild cognitive impairment; HC: healthy control

The y-axis is the proportion of cohesive words (range: 9.76~28.84(%)) of each participant; the x-axis indicates the individual numbers of aMCI patients (1~30), naMCI patients (1~22), and the HC (1~21).

**Figure 3** Scatter plot of propositional density in aMCI, naMCI, and HC

aMCI: amnestic mild cognitive impairment; naMCI: non-amnestic mild cognitive impairment; HC: healthy control

The y-axis is propositional density (range: 33.34~92.96(%)) of each participant; the x-axis indicates the individual numbers of aMCI patients (1~30), naMCI patients (1~22), and the HC (1~21).

**Figure 4** Scatter plot of the proportion of pauses in aMCI, naMCI, and HC

aMCI: amnestic mild cognitive impairment; naMCI: non-amnestic mild cognitive impairment; HC: healthy control

The y-axis is the proportion of pauses (range: 0~38.75(%)) of each participants; the x-axis indicates the individual numbers of aMCI patients (1~30), naMCI patients (1~22), and the HC (1~21).
